# Low value of whole-body dual-modality [18f]fluorodeoxyglucose positron emission tomography/computed tomography in primary staging of stage I–II nasopharyngeal carcinoma: a nest case-control study

**DOI:** 10.1007/s00330-020-07478-1

**Published:** 2021-01-08

**Authors:** Bei-Bei Xiao, Qiu-Yan Chen, Xue-Song Sun, Ji-Bin Li, Dong-hua Luo, Rui Sun, Da-Feng Lin, Xu Zhang, Wei Fan, Xiao-Fei Lv, Lu-Jun Han, Yue-Feng Wen, Li Yuan, Shan-Shan Guo, Li-Ting Liu, Sai-Lan Liu, Qing-Nan Tang, Yu-Jing Liang, Xiao-Yun Li, Chao Lin, Ling Guo, Hai-Qiang Mai, Lin-Quan Tang

**Affiliations:** 1 Sun Yat-sen University Cancer Center, State Key Laboratory of Oncology in South China, Collaborative Innovation Center for Cancer Medicine, Guangdong Key Laboratory of Nasopharyngeal Carcinoma Diagnosis and Therapy, 651 Dongfeng Road East, Guangzhou, 510060 People’s Republic of China; 2 Department of Nasopharyngeal Carcinoma, Sun Yat-sen University Cancer Center, 651 Dongfeng Road East, Guangzhou, 510060 People’s Republic of China; 3 Department of Clinical Research, Sun Yat-sen University Cancer Center, 651 Dongfeng Road East, Guangzhou, 510060 People’s Republic of China; 4 Department of Nuclear Medicine, Sun Yat-sen University Cancer Center, 651 Dongfeng Road East, Guangzhou, 510060 People’s Republic of China; 5 Department of Medical Imaging, Sun Yat-sen University Cancer Center, 651 Dongfeng Road East, Guangzhou, 510060 People’s Republic of China

**Keywords:** Nasopharyngeal carcinoma, PET/CT, MRI, Neoplasm staging

## Abstract

**Objectives:**

The value of using PET/CT for staging of stage I–II NPC remains unclear. Hence, we aimed to investigate the survival benefit of PET/CT for staging of early-stage NPC before radical therapy.

**Methods:**

A total of 1003 patients with pathologically confirmed NPC of stages I–II were consecutively enrolled. Among them, 218 patients underwent both PET/CT and conventional workup ([CWU], head-and-neck MRI, chest radiograph, liver ultrasound, bone scintigraphy) before treatment. The remaining 785 patients only underwent CWU. The standard of truth (SOT) for lymph node metastasis was defined by the change of size according to follow-up MRI. The diagnostic efficacies were compared in 218 patients who underwent both PET/CT and CWU. After covariate adjustment using propensity scoring, a cohort of 872 patients (218 with and 654 without pre-treatment PET/CT) was included. The primary outcome was overall survival based on intention to treat.

**Results:**

Retropharyngeal lymph nodes were metastatic based on follow-up MRI in 79 cases. PET/CT was significantly less sensitive than MRI in detecting retropharyngeal lymph node lesions (72.2% [62.3–82.1] vs. 91.1% [84.8–97.4], *p* = 0.004). Neck lymph nodes were metastatic in 89 cases and PET/CT was more sensitive than MRI (96.6% [92.8–100.0] vs. 76.4% [67.6–85.2], *p* < 0.001). In the survival analyses, there was no association between pre-treatment PET/CT use and improved overall survival, progression-free survival, local relapse-free survival, regional relapse-free survival, and distant metastasis-free survival.

**Conclusions:**

This study showed PET/CT is of little value for staging of stage I–II NPC patients at initial imaging.

**Key Points:**

• *PET/CT was more sensitive than MRI in detecting neck lymph node lesions whereas it was significantly less sensitive than MRI in detecting retropharyngeal lymph node lesions.*

• *No association existed between pre-treatment PET/CT use and improved survival in stage I–II NPC patients.*

**Supplementary Information:**

The online version contains supplementary material available at 10.1007/s00330-020-07478-1.

## Introduction

Nasopharyngeal carcinoma (NPC) is a malignant tumor with a worldwide incidence of 0.5–1.0/100,000 per year, but occurs with a much higher incidence rate in southern China and Southeast Asia, in which it is endemic with an incidence rate of 30/100,000 persons per year [1, 2]. The examinations for the initial staging of NPC comprise magnetic resonance imaging (MRI) of the head and neck, chest radiograph, liver ultrasound, bone scintigraphy (conventional workup [CWU]), and/or [18F]fluorodeoxyglucose positron emission tomography/computed tomography (PET/CT). The value of PET/CT and CWU at the initial staging of NPC has been extensively studied. Previous studies have reported about the superiority of MRI in detecting local tumor involvement and RLN (retropharyngeal lymph node) metastasis while PET/CT is advantageous in demonstrating CLN (cervical lymph node) metastasis and occult distant metastasis [3–5]. Besides, Lin et al demonstrated that PET/CT was useful in revealing occult distant metastases among NPC patients with advanced node disease [5–7]. Moreover, our previous study demonstrated that the NPC patients with stage N2–3 and Epstein–Barr virus (EBV) DNA ≥ 4000 copies/mL would benefit more from PET/CT to detect distant metastasis [8]. With regard to local recurrence and residual NPC, the PET/CT was superior to MRI in diagnostic value based on the results of a meta-analysis [9]. Thus, it can be concluded that PET/CT is very important in the primary staging of NPC patients.

However, in relation to early-stage (stage I–II) NPC, there were few studies concerning the clinical benefit of PET/CT in this specific population. Besides, most studies focusing on the accuracy of PET/CT and CWU in NPC lacked long-term follow-up [3]. Furthermore, the 2019 guidelines of National Comprehensive Cancer Network (NCCN) recommended that PET/CT should be considered for NPC patients to detect distant metastases [10], evidence on clinical benefit of PET/CT in early-stage NPC is still lacking and worth studying. Therefore, we conducted this longitudinal cohort study to investigate the clinical benefit of PET/CT in early-stage NPC patients.

## Materials and methods

### Patients

From January 2007 to December 2014, 1003 consecutive patients histologically diagnosed with stage I–II NPC based on CWU in Sun Yat-sen University Cancer Center were enrolled in this study. Among them, 218 patients underwent both PET/CT and CWU before treatment and the remaining 795 patients only underwent CWU. The eligibility criteria were as follows: (1) histologically diagnosed WHO type II or III NPC; (2) clinical stage I or II (American Joint Committee on Cancer [AJCC] 8th staging system) based on CWU; (3) no history of a second malignant tumor; and (4) Eastern Cooperative Oncology Group (ECOG) performance score ≤ 2. All examinations were performed within 2 weeks, and all patients were restaged based on the 8th AJCC staging manual. The study was approved by the clinical research ethics committee of Sun Yat-sen University Cancer Center and informed consent was obtained from all participants.

Considering potential confounders, propensity score matching (PSM) was performed among 1003 patients grouped by the examination method (PET/CT + CWU or CWU alone), and a well-balanced cohort including 218 patients (PET/CT plus CWU) and 654 patients (CWU only) was identified.

### PET/CT and MRI imaging

PET/CT and MRI imaging information were included in the supplemental material.

### Image interpretation

Head-and-neck MRI imaging was interpreted by two experienced radiologists. Regarding the neck and retropharyngeal lymph nodes on MRI, a diagnosis of metastatic lymph node was made if the presence of necrosis or extracapsular spread or cluster lymph nodes (≥ 3) was detected. As for the size of lymph node, if the shortest axial diameter of the retropharyngeal node was 5 mm or greater, the lymph node was considered positive [11]. Similarly, the 10-mm shortest axial diameter was the borderline size of positive CLN [12]. Two experienced nuclear medicine physicians interpreted PET/CT images without knowing patients’ MRI results and clinical information [7, 13]. Lymph nodes were identified as positive if they showed significantly different tracer uptake compared to the background [14]. Any discrepancy was discussed by the research team and reached a consensus. The PET/CT results were added to CWU findings to make the final decision-making. Whether PET/CT results modified patients’ clinical classification and subsequent treatment strategies were recorded.

### The standard of truth

For patients in which PET/CT detected suspicious metastatic lesions, the validation was determined by assessing the histopathology or subsequent imaging examinations. The suspicious metastatic lesion was considered positive only if positive pathology result was obtained or at least two imaging examinations have positive results or suspicious lesions have progression within 1-year follow-up.

All patients were checked every 3 months within 12 months after initial diagnosis [4, 13]. According to the MRI imaging performed at 3 months after the entire treatment, a retropharyngeal or cervical lymph node lesion was determined as true positive if it was evaluated as showing partial response (PR), complete response (CR), or progressive disease (PD) based on the Response Evaluation Criteria in Solid Tumors [15]. If the lymph node showed stable disease (SD) at 3 months after treatment, regular follow-up examinations for 1 year were compulsory. If the lymph node was found to enlarge by 120% or more in size within 1 year of follow-up, it was considered a true-positive lymph node, otherwise it was identified as true negative [16]. Distant metastasis detected within 1 year of follow-up was defined as proof of primary metastasis and false-negative screening.

### Treatment and follow-up

All eligible patients were treated with intensity-modulated radiation therapy (IMRT). Target volumes were delineated slice-by-slice on treatment planning CT scans by using an individualized delineation protocol. Generally, a cumulative dose of 66 ~ 72 Gy was administered for the planning target volume of the primary gross tumor volume (GTVnx) and the GTV of the involved lymph nodes (GTVnd), with 1.8–2.1 Gy per fraction and five daily fractions per week. All potential metastatic CLN drainage areas were prophylactically irradiated with at least 50 Gy. Among 1003 patients, 426 patients were assigned to radiotherapy (RT) only, 378 patients received concurrent chemoradiotherapy (CCRT), 82 patients underwent the combination of induction chemotherapy (ICT) and RT, and 117 patients received ICT plus CCRT. Among 218 patients who underwent PET/CT, 90 patients were assigned to radiotherapy (RT) alone, 78 patients received concurrent chemoradiotherapy (CCRT), 19 patients underwent induction chemotherapy (ICT) plus RT, and 31 patients received ICT and CCRT.

After the completion of treatment, follow-up examinations were performed every 3 months in the first 2 years, every 6 months in the following 3 years, and once a year thereafter [10]. Comprehensive physical examinations including nasopharyngoscopy, serum EBV DNA measurement, MRI of the head and neck, chest radiography, abdominal sonography, and bone scan were performed at each follow-up visit. If the tumor relapsed or distant metastasis was suspected, biopsy or targeted imaging methods were used to differentiate benign from malignant lesions.

### Statistical analysis

PSM and covariate adjustment were performed using appropriate R software package. The independent variables thought to possibly affect study outcomes included age, gender, smoking, EBV DNA level, T category, N category, clinical stage, and treatment modality. The dependent variable was whether PET/CT had been performed. Finally, PSM with a matching ratio of 3 identified a cohort of 872 patients.

Survival rates were compared using Kaplan–Meier analysis with log-rank tests. Overall survival (OS), i.e., the time from diagnosis to the date of death from any reason or the date of the last follow-up, was the primary study outcome [17]. Progression-free survival (PFS) was the time from diagnosis to cancer progression or death from any reason. Local relapse-free survival (LRFS) or regional recurrence-free survival (RRFS) was calculated as the interval from diagnosis to local relapse or death from any reason, regional recurrence or death from any reason, respectively, while locoregional relapse-free survival (LRRFS) was calculated as the time from diagnosis to locoregional reoccurrence or death from any reason. Distant metastasis-free survival (DMFS) referred to the time from diagnosis to the date of distant metastasis or death from any reason. Patients lost to follow-up or alive without distant metastasis or locoregional recurrence at the last follow-up visit had their data censored. We estimated and compared the 5-year survival rates of PET/CT + CWU and CWU groups. Multivariable Cox regression models with an enter method were built to identify dependent prognostic factors of early-stage NPC using subsets of data after PSM.

McNemar’s paired-sample test or *χ*^2^ test was applied to determine whether the sensitivity, specificity, positive predictive value, and negative predictive value of PET/CT and MRI in diagnosing lymph node metastasis were significantly different in the PET/CT + CWU cohort.

Statistical analyses were performed with R software package (Version 3.5.2, http://www.r-project.org/) and SPSS 25 (IBM). All statistical tests were two-sided, and *p* values of < 0.05 were considered significant.

## Results

### Staging discrepancies between PET/CT and CWU

**Fig. 1:**
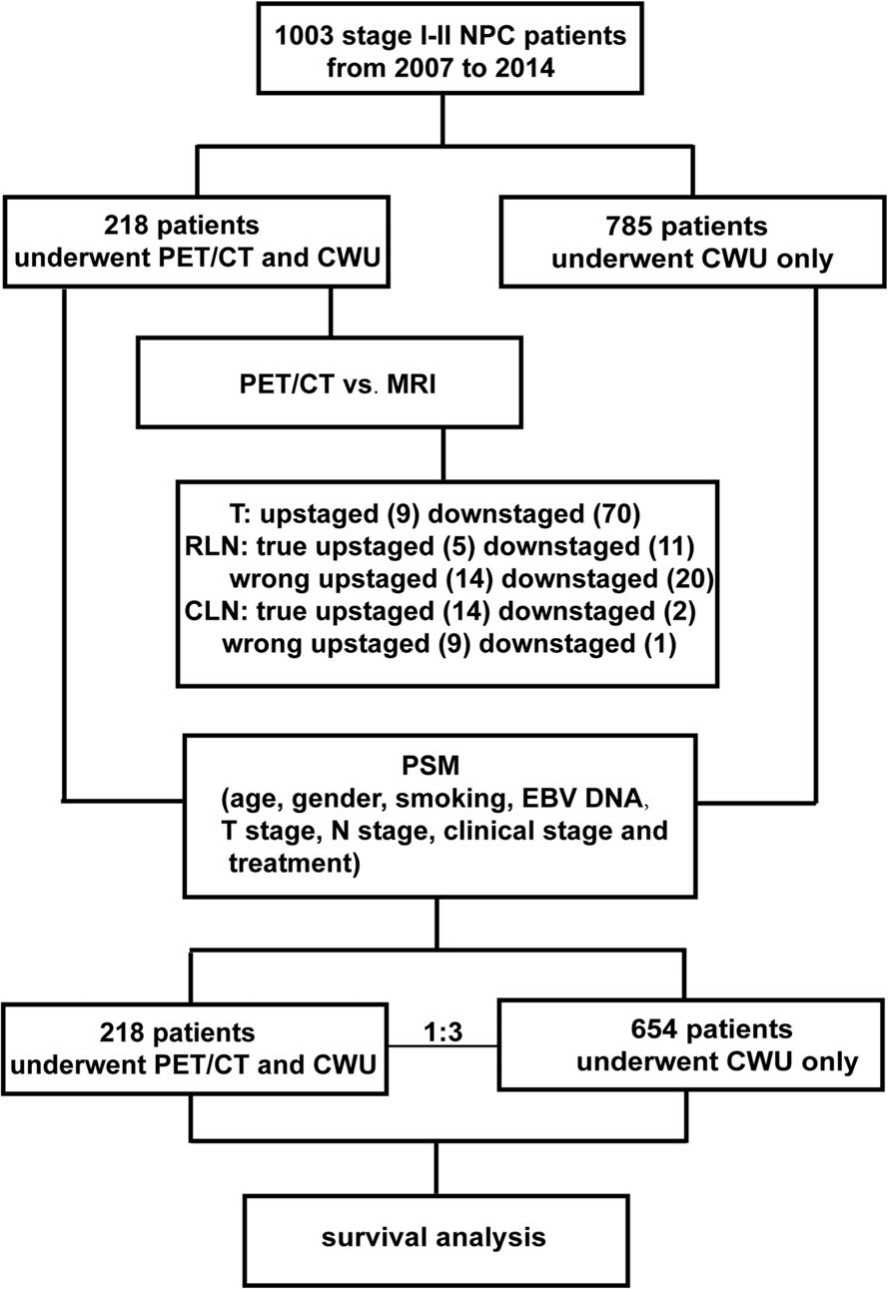
Flowchart of this study

**Fig. 2:**
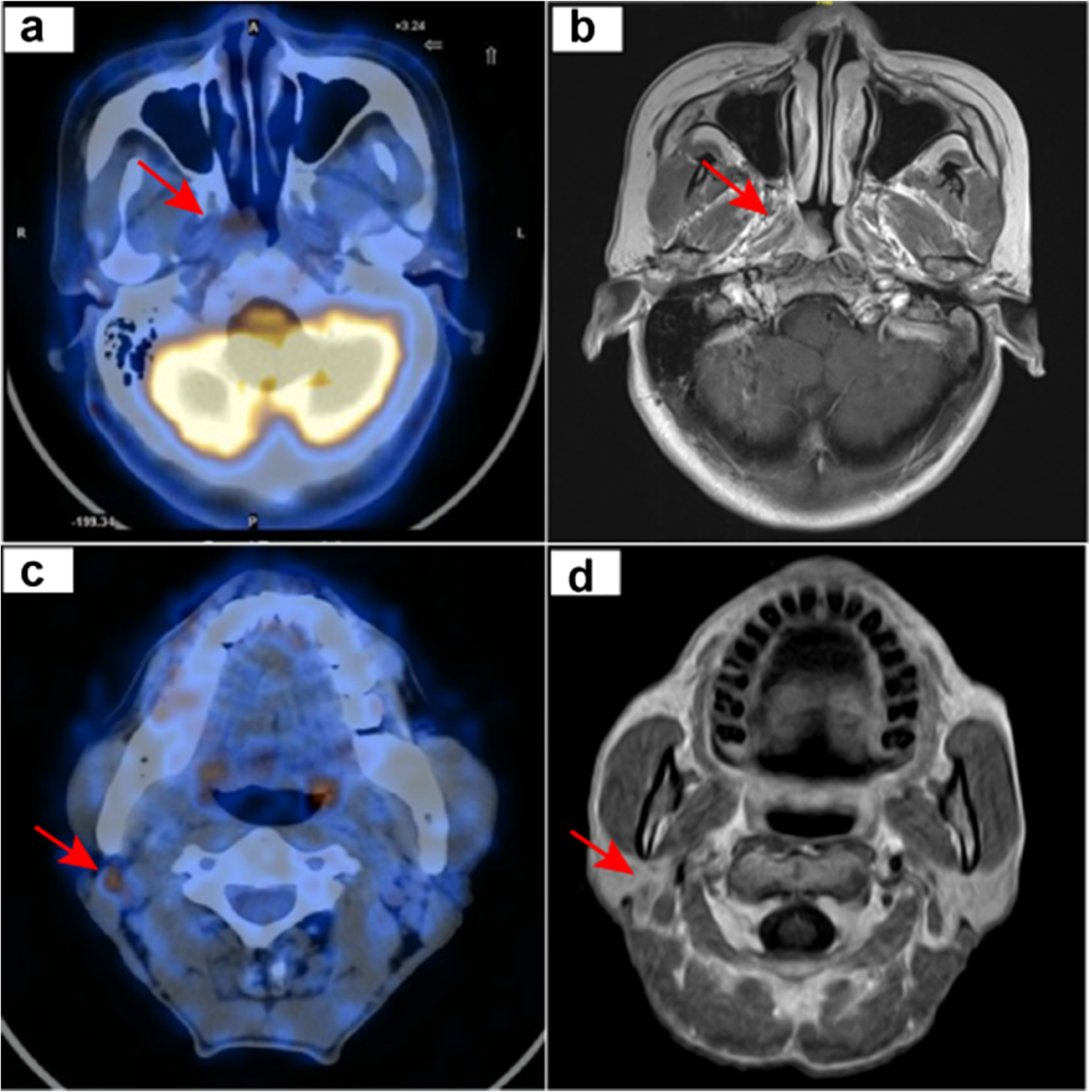
T- and N-staging discrepancies between PET/CT and head-and-neck MRI. (**a**, **b**) Female, 59 years old, T2N0M0 NPC. PET/CT image (left). Contrast-enhanced T1-weighted MRI image (right). PET/CT did not detect the parapharyngeal lesion that was found by MRI (red arrow). (**c**, **d**) Male, 57 years old, T2N1M0 NPC. PET/CT image (left). Contrast-enhanced T1-weighted MRI image (right). PET/CT distinguished positive neck lymph node (red arrow) while it was regarded as a morphological benign lymph node in MRI

In the PET/CT + CWU group, 133 and 85 patients were respectively diagnosed as category T1 and T2 based on MRI (Fig. 1). PET/CT altered the T category diagnosis in 79 patients, with nine patients being upstaged and 70 downstaged due to the discrepancy in determining parapharyngeal space involvement (Fig. 2a, b). As for N category, 101 and 117 patients were classified as N0 and N1, respectively. PET/CT upstaged 28 patients and downstaged 24 patients.

A total of 218 patients primarily underwent both PET/CT and CWU and showed no distant metastasis. PET/CT staging did not change patients’ major treatment strategy. Although PET/CT modified the planned target volume and dose in 25 (11.5%) patients since their N categories were truly changed according to the standard of truth (SOT), with 15 upstaged and 10 downstaged, the treatment plans of 193 (88.5%) patients were not changed. Among those 193 patients, both imaging tests achieved consistent results in 105 (54.4%) patients and PET/CT wrongly upstaged or downstaged MRI-based N category in 27 patients according to the SOT. Besides, PET/CT upstaged and downstaged MRI-based T category in 9 and 70 patients, respectively, without knowing if it was changed truly because of lack for SOT. Given that PET/CT was inferior to MRI in detecting soft tissue lesions, therefore, the T staging changed by PET/CT did not obviously influence patients’ treatment strategies.

### Retropharyngeal lymph node metastasis

**Fig. 3:**
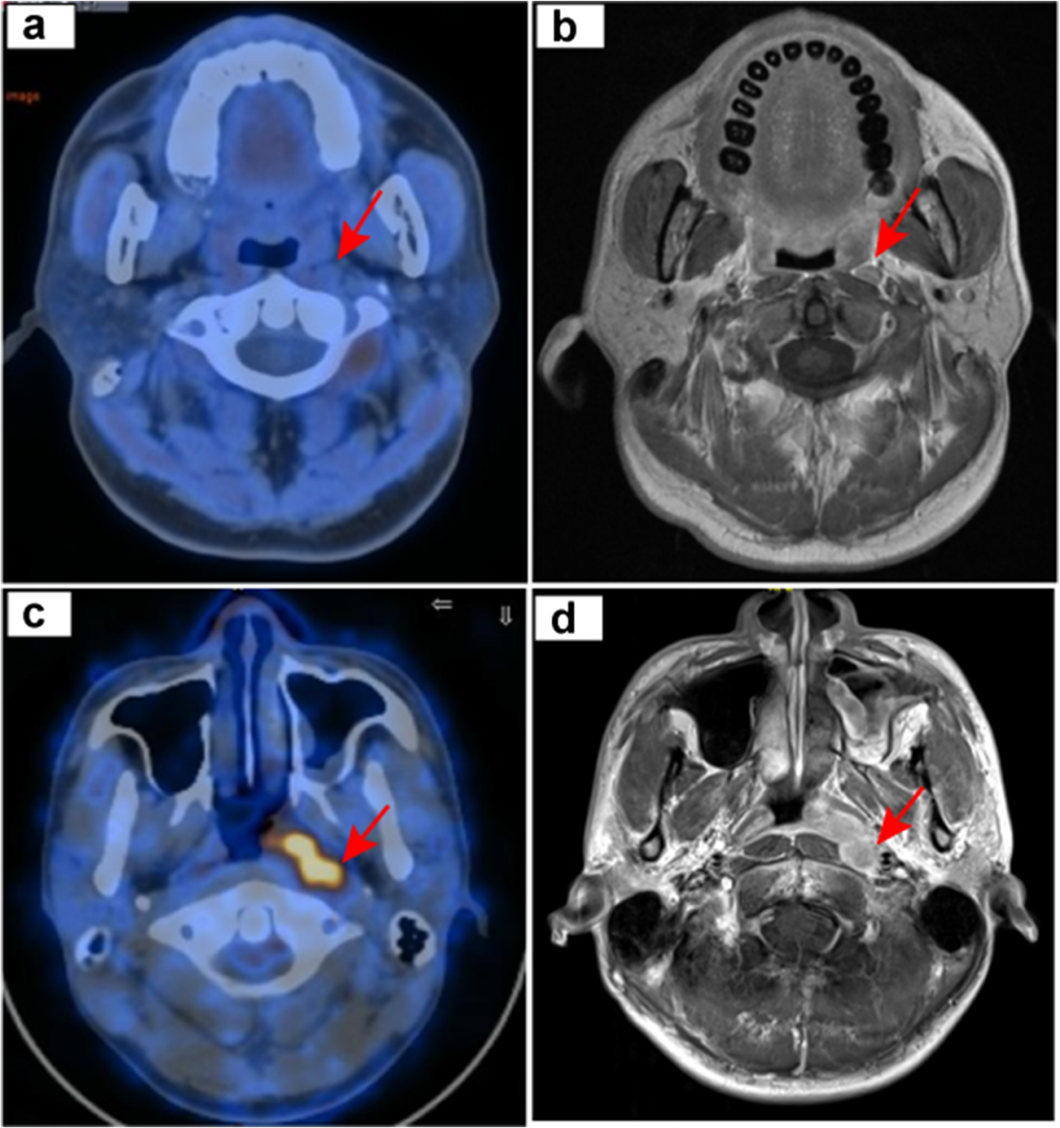
Detecting discrepancies of retropharyngeal and neck lymph nodes between PET/CT and head-and-neck MRI. (**a**, **b**) Male, 45 years old, T2N1M0 NPC. PET/CT image (left). Contrast-enhanced T1-weighted MRI image (right). Metastatic retropharyngeal lymph node was detected by MRI (red arrow) but not PET/CT. (**c**, **d**) Male, 50 years old, T2N1M0 NPC. PET/CT image (left). Contrast-enhanced T1-weighted MRI image (right). Metastatic retropharyngeal lymph node was obvious on MRI (red arrow), while it disappeared on PET/CT because of the shadow of the primary lesion

According to the SOT, RLNs were identified as metastatic in 79 cases, of which 57 were detected by PET/CT and 72 by MRI. PET/CT was significantly less sensitive than MRI in detecting RLN lesions (72.2% [62.3–82.1] vs. 91.1% [84.8–97.4], *p* = 0.004) (Figure S1a). The specificity, positive predictive value (PPV), and negative predictive value (NPV) of PET/CT and MRI for detecting RLN lesions were 88.5% vs. 90.6%, 78.1% vs. 84.7%, and 84.8% vs. 94.7%, respectively (Table 1). Fifty (22.9%) patients presented with inconsistent results between PET/CT and MRI. MRI correctly diagnosed 31 cases out of 50 patients. PET/CT missed truly metastatic RLNs in 20 patients and showed false-positive results in 14 patients (Fig. 3). Among the 16 patients with truly metastatic LNs who were diagnosed correctly by PET/CT, five had small RLNs with a shortest axial diameter less than 5 mm but with high FDG uptake. All doubtful LNs were treated with radical radiotherapy without the acquirement of retropharyngeal nodal biopsy due to the anatomical inaccessibility.

**Table 1:** The sensitivity, specificity, PPV, and NPV of PET/CT and MRI in diagnosing RLNs and CLNs in 218 patients

Site	Test	No. of patients	FN	TP	TN	FP	Sensitivity			Specificity			PPV			NPV		
							%	95%CI (%)	*p*	%	95%CI (%)	*p*	%	95%CI (%)	*p*	%	95%CI (%)	*p*
Retropharyngeal lymph node metastasis	PET/CT	79	22	57	123	16	72.2 (57/79)	(62.3–82.1)		88.5 (123/139)	(83.2–93.8)		78.1 (57/73)	(68.6–87.6)		84.8 (123/145)	(79.0–90.6)	
	MRI		7	72	126	13	91.1 (72/79)	(84.8–97.4)		90.6 (126/139)	(85.7–95.5)		84.7 (72/85)	(77.0–92.4)		94.7 (126/133)	(90.9–98.5)	
	PET/CT vs MRI								0.004			0.690			0.284			0.007
Neck lymph node metastasis	PET/CT	89	3	86	94	35	96.6 (86/89)	(92.8–1.0)		72.9 (94/129)	(65.2–80.6)		71.1 (86/121)	(63.0–79.2)		96.9 (94/97)	(93.5–1.0)	
	MRI		21	68	124	5	76.4 (68/89)	(67.6–85.2)		96.1 (124/129)	(92.8–99.4)		93.2 (68/73)	(87.4–99.0)		85.5 (124/145)	(79.8–91.2)	
	PET/CT vs MRI								< 0.001			< 0.001			< 0.001			0.004

Statistical comparisons were made using McNemar’s paired-sample test, *χ*^2^ test

*Abbreviations*: *RLN*, retropharyngeal lymph node; *CLN*, cervical lymph node; *CWU*, conventional workup; *PET/CT*, 18F_fluorodeoxyglucose positron emission tomography/computed tomography; *FN*, false negative; *TP*, true positive; *TN*, true negative; *FP*, false positive; *PPV*, positive predictive value; *NPV*, negative predictive value

### Cervical lymph node and distant metastasis

CLNs at initial diagnosis in 89 patients were confirmed to be metastatic based on SOT. Among them, 86 patients and 68 patients were correctly diagnosed by PET/CT and MRI accordingly. PET/CT was more sensitive than MRI (96.6% [92.8–100.0] vs. 76.4% [67.6–85.2], *p* < 0.001) (Figure S1b). The comparisons of specificity, PPV, and NPV between PET/CT and MRI for detecting CLNs were 72.9% vs. 96.1%, 71.1% vs. 93.2%, and 96.9% vs. 85.5%, respectively (Table 1). Among 26 (11.9%) patients showing inconsistent CLN results, PET/CT presented true-positive findings in 14 patients with morphologically benign LNs in MRI (Fig. 2c, d) and true-negative findings in two patients with morphologically malignant lymph nodes on MRI. Nonetheless, PET/CT yielded wrong results in ten patients, which included false-positive results in nine patients and false-negative result in one patient. In terms of distant metastasis, all 218 patients underwent both PET/CT and CWU before initial treatment and no distant metastasis was detected. However, one patient developed liver metastasis within 1 year after diagnosis.

### Survival analysis in the PET/CT and CWU groups

The PSM cohort included 218 NPC patients who underwent both PET/CT and CWU and 654 patients who only underwent CWU. No imbalanced covariate existed between these two groups. The demographics and clinical characteristics of patients were shown in Table 2. The median follow-up period in the PET/CT (PETCT + CWU) group was 63.0 (interquartile range [IQR], 55.7–74.2) months. During the follow-up, four (1.8%) patients died, of which three (1.4%) died of disease progression and one (0.5%) died from other reasons. Nine (4.1%) patients showed disease progression, of which 7 (3.2%) developed local occurrence, 3 (1.4%) patients developed regional occurrence, and 5 (2.3%) showed distant metastasis. In the CWU group, the median follow-up period was 67.3 months (IQR, 55.6–83.3 months). Twenty-six (4.0%) patients died, of which 22 (3.4%) patients’ deaths resulted from NPC progression and 4 (0.6%) patients died from other reasons. In addition, 60 (9.2%) patients developed disease progression, including 23 (3.5%) cases of local occurrence, 17 (2.6%) cases of regional occurrence, and 30 (4.6%) cases of distant metastases.

**Table 2:** Baseline characteristics of observational dataset and PSM dataset (*n* = 872)

Characteristic	Observational dataset (*n* = 1003)			PSM dataset (*n* = 872)		
	PET/CT + CWU (*n* = 218)	CWU (*n* = 785)	*p*	PET/CT + CWU (*n* = 218)	CWU (*n* = 654)	*p*
Age (yr)			0.167			0.334
Median(range)	44.5 (35–51)	47 (20–81)		44.5 (35–51)	46 (39–54)	
≤ 45	119 (54.6)	387 (49.3)		119 (54.6)	342 (52.3)	
> 45	99 (45.4)	398 (50.7)		99 (45.4)	312 (47.7)	
Gender			0.083			0.743
Female	47 (21.6)	215 (27.4)		47 (21.6)	148 (22.6)	
Male	171 (78.4)	570 (72.6)		171 (78.4)	506 (77.4)	
T stage			0.020			0.810
T1	133 (61.0)	409 (52.1)		133 (61.0)	393 (60.1)	
T2	85 (39.0)	376 (47.9)		85 (39.0)	261 (39.9)	
N stage			0.992			0.906
N0	101 (46.3)	364 (46.4)		101 (46.3)	306 (46.8)	
N1	117 (53.7)	421 (53.6)		117 (53.7)	348 (53.2)	
Clinical stage			0.175			1.000
I	74 (33.9)	229 (29.2)		37 (17.6)	125 (19.8)	
II	144 (66.1)	556 (70.8)		173 (82.4)	505 (80.2)	
EBV DNA			0.012			0.139
< 4000	168 (77.1)	662 (84.3)		168 (77.1)	534 (81.7)	
≥ 4000	50 (22.9)	123 (15.7)		50 (22.9)	120 (11.3)	
Smoking			0.722			0.445
No	156 (71.6)	552 (70.3)		156 (71.6)	450 (68.8)	
Yes	62 (28.4)	233 (29.7)		62 (28.4)	204 (31.2)	
Treatment			0.568			0.334
RT	90(55.5)	336 (42.8)		90 (41.3)	288 (44.0)	
CCRT	78 (45.5)	300 (38.2)		78 (35.8)	251 (38.4)	
ICT + RT	19 (8.7)	63 (8.0)		19 (8.7)	49 (7.5)	
ICT + CCRT	31 (14.2)	86 (11.0)		31 (14.2)	66 (10.1)	

*p* value < 0.05 indicates a statistically significant difference

*Abbreviations*: *yr*, year; *EBV*, Epstein–Barr virus; *RT*, radiotherapy; *CCRT*, concurrent chemoradiotherapy; *ICT*, induction chemotherapy

**Fig. 4:**
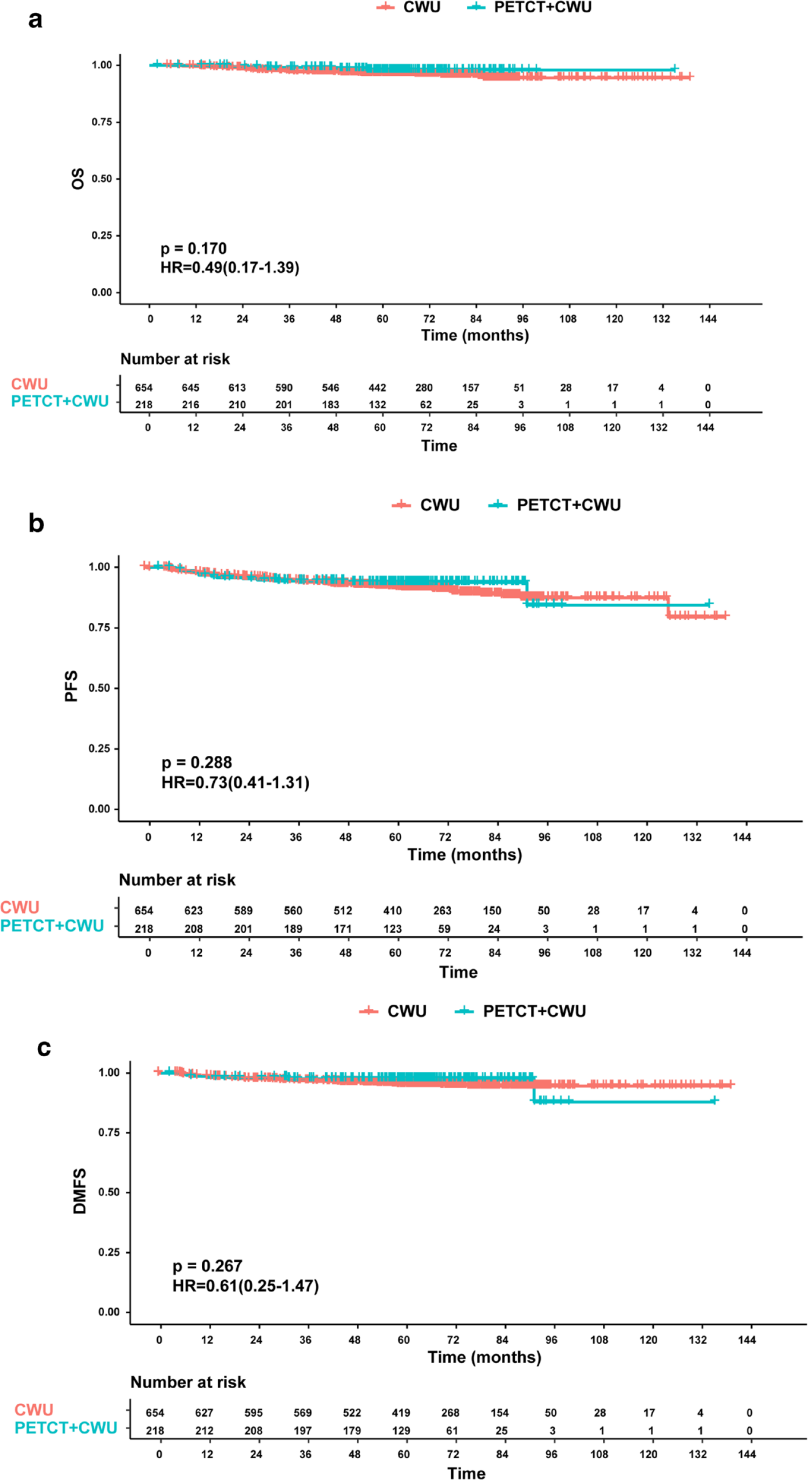
Kaplan–Meier survival curves for 872 stage I–II NPC patients stratified by the implementation of PET/CT: (**a**) overall survival, (**b**) progression-free survival, (**c**) distant metastasis-free survival

**Fig. 5:**
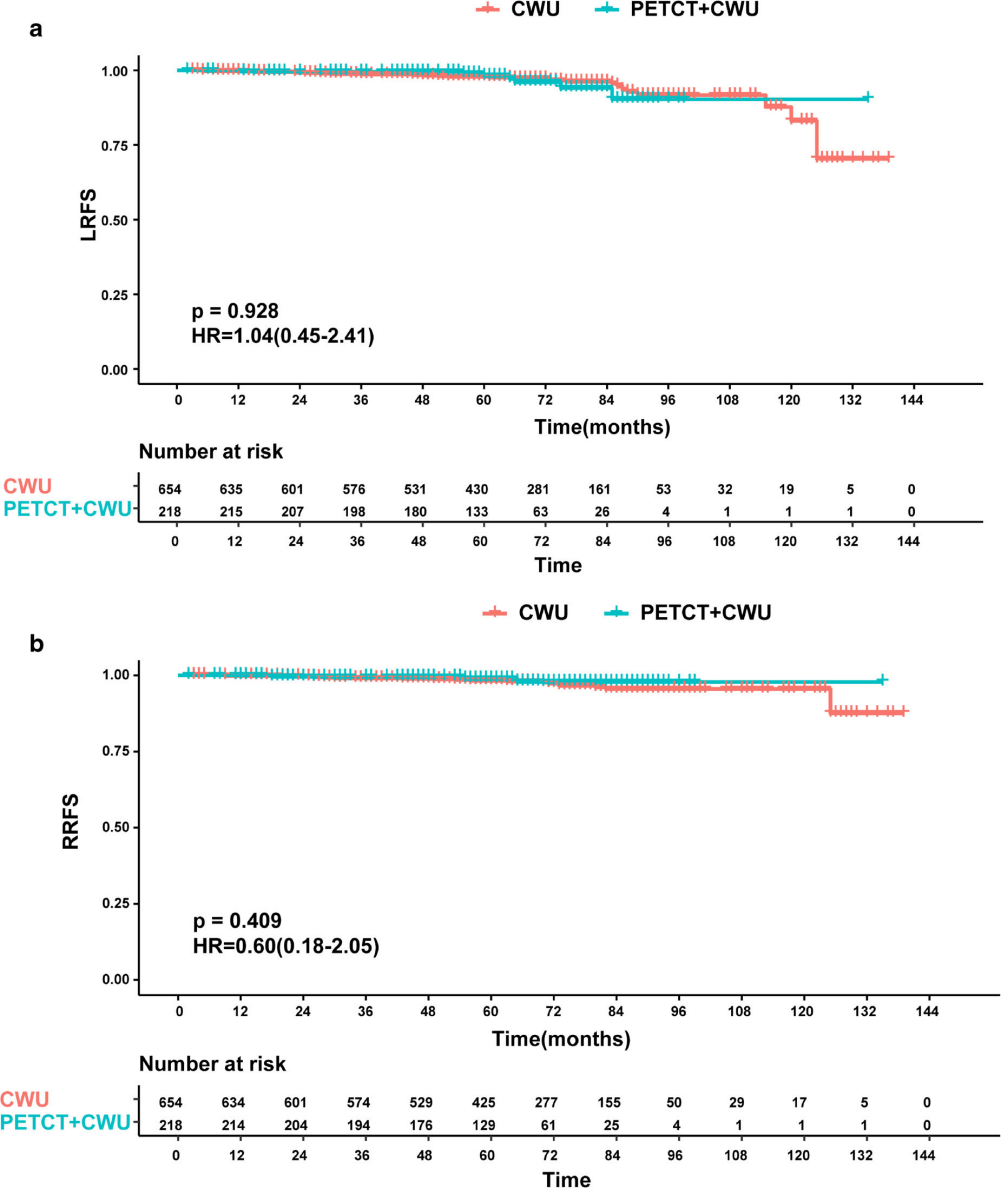
Kaplan–Meier survival curves for 872 stage I–II NPC patients stratified by the implementation of PET/CT: (**a**) local relapse-free survival, (**b**) regional relapse-free survival

The 5-year OS rate in the PET/CT group was 97.9% (95% CI 96.9–98.9) in comparison with 96.4% (95% CI 95.6–97.2) in the CWU group (*p* = 0.170) (Fig. 4a). The 5-year LRFS and RRFS rates were 98.9% and 98.4% in the PET/CT cohort, and 98.2% and 97.8% in the CWU cohort (*p* = 0.928 and 0.409, respectively) (Fig. 5). Similarly, the survival outcomes showed no significant difference between the PET/CT and CWU groups in terms of PFS and DMFS (5-year PFS: 93.8% vs. 91.7%, *p* = 0.288; 5-year DMFS: 97.7%vs. 95.3%, *p* = 0.267) (Fig. 4b, c).

### Multivariate analysis

In multivariate analysis, age, gender, T category, N category, clinical stage, smoking, EBV DNA level, treatment modality, and PET/CT application were considered as covariates and entered into Cox’s proportional hazards model (*n* = 872). As shown in Table 3, the use of PET/CT was not associated with improved OS (*p* = 0.262), PFS (*p* = 0.196), LRRFS (*p* = 0.526), and DMFS (*p* = 0.182) in early-stage NPC. Age (HR = 3.16, 95% CI 1.44–6.92, *p* = 0.004) and N category (HR = 4.14, 95% CI 1.67–10.16, *p* = 0.002) were evaluated as independent prognostic factors for OS. Two covariates were identified as independent prognostic factors for PFS, namely, N category (HR = 2.44, 95% CI 1.48–4.00, *p* < 0.001) and EBV DNA level (HR = 1.74, 95% CI 1.01–2.97, *p* = 0.042). N category (HR = 3.50, 95% CI 1.73–7.07, *p* < 0.001) and EBV DNA level (HR = 3.55, 95% CI 1.84–6.85, *p* < 0.001) were found to be independent prognostic factors for LRRFS and DMFS, respectively.

**Table 3:** Multivariable Cox models for PSM dataset (*n* = 872)

	PFS		OS		LRRFS		DMFS	
	HR (95% CI)	*p* value	HR (95% CI)	*p* value	HR (95% CI)	*p* value	HR (95% CI)	*p* value
Age		NS		0.004		NS		NS
≤ 45			Reference					
> 45			3.16(1.44–6.92)					
N stage		< 0.001		0.002		< 0.001		NS
N0	Reference		Reference		Reference			
N1	2.44(1.48–4.00)		4.14(1.67–10.16)		3.50(1.73–7.07)			
EBV DNA		0.042		NS				< 0.001
< 4000	Reference						Reference	
≥ 4000	1.74(1.01–2.97)						3.55(1.84–6.85)	
PET/CT obtained		0.262		0.196		0.526		0.182
Yes	Reference		Reference		Reference		Reference	
No	0.72(0.40–1.28)		0.56(0.23–1.35)		0.79(0.38–1.64)		0.49(0.17–1.40)	

*p* value < 0.05 indicates a statistically significant difference

*Abbreviations*: *PFS*, progression-free survival; *OS*, overall survival; *LRRFS*, locoregional relapse-free survival; *DMFS*, distant metastasis-free survival

## Discussion

Although many studies have clarified the significant value of PET/CT in the initial imaging of patients with locoregional advanced NPC [3, 18], this technique may not play an equally significant role for stage I–II NPC in actual clinical practice. This is a nest case-control study to evaluate the clinical benefit of PET/CT in early-stage NPC over initial imaging with a long-term follow-up time. Consistent with previous studies [6, 19–21], we verified that PET/CT was superior to MRI in detecting CLN metastases but inferior in diagnosing RLN involvement. Nevertheless, we also found that the survival outcomes of NPC patients in survival analysis did not differ depending on whether PET/CT was performed.

In the present study, significant discrepancies existed in PET/CT and MRI in assessing the involvement of parapharyngeal space and the RLNs and CLNs. On the basis of MRI diagnosis, PET/CT altered T staging in 36.2% (79) patients, with 4.1% (9) being upstaged and 32.1% (70) being downstaged. Our results implied that PET/CT was not as sensitive as MRI in detecting parapharyngeal space invasion, which was concordant with some previous studies [3]. This might be an outcome of the spillover effect of PET/CT and low FDG uptake in early-stage NPC. In terms of 9 patients with upstaged T category by PET/CT imaging but did not treat based on it, no patients developed local recurrence until the last follow-up. This might further imply the false-positive results of PET/CT. In this study, PET/CT staging did not change patients’ major treatment strategy. We assumed that the use of PET/CT might change some patients’ chemotherapy regimens but this is a retrospective study so that we could not be able to obtain the exact percentage. However, according to previous studies and our data, PET/CT is inferior to MRI in detecting soft tissue lesions. Moreover, the small portion changes of treatment regimens did not result in patients’ survival difference based on our long-term follow-up. Therefore, we concluded that PET/CT staging did not change early-stage patients’ major treatment strategy.

The fact that MRI is superior to PET/CT for detecting metastatic RLNs has been verified by a previous study [22]; the same conclusion was drawn in the current study. Among the 79 inconsistent results obtained from PET/CT and MRI, MRI yielded correct diagnoses in 72 patients. Besides, we found that PET/CT was inclined to miss malignant RLNs because they were nearly connected with an adjacent primary tumor in many cases.

CLN metastasis plays a critical role in NPC due to its high incidence rate and independent prognostic value. It is also of vital importance in staging and prognostic classification of early-stage NPC [23, 24]. For the cases showing inconsistent lymph node results between PET/CT and MRI, we found that PET/CT was more accurate than MRI in diagnosing CLN lesion. Although the functional imaging ability of PET/CT facilitates detection of CLN metastasis, it simultaneously caused difficulties in discriminating metastatic CLNs from inflammatory lymph nodes.

Distant metastasis is one of the most crucial factors guiding treatment planning in oncology which necessitates the accurate diagnosis [25]. Yen [13] demonstrated that the N category of NPC strongly influenced the possibility of distant metastasis. In our study, CWU and PET/CT did not detect any abnormality of primary distant metastasis at initial imaging, and only one patient developed liver metastasis within 1 year. Considering the low incidence rate of distant metastasis among parents with early-stage NPC, we inferred that PET/CT may not offer benefits over CWU for detecting distant metastasis in early-stage NPC. Although we found that PET/CT was superior to MRI in detecting CLN metastases, no association was observed between survival benefit and the application of pre-treatment PET/CT. It might be explained by the fact that PET/CT’s use did not change patients’ main treatment courses and all patients received prophylactic cervical irradiation. Changing the radiotherapy strategies due to N category might affect the quality of life; however, we could not be able to make an analysis. Because this is a retrospective study, we could not be able to compare patients’ quality of life brought by use of PET/CT with patients who did not.

Our study had several limitations. Firstly, the SOT for lymph node metastasis in this study was defined by the change of size according to MRI follow-up instead of pathology which was regarded as the gold standard for diagnosis. Secondly, the lesion of parapharyngeal space that lacked SOT was due to the difficulty in obtaining histopathology result. Thirdly, we could not be able to obtain the effect of radiotherapy strategies on patients’ life quality because the retrospective study and prospective study need to be carried out. Finally, all patients were from a single center in an epidemic area which prompted further verification from other institutions.

In conclusion, this study shows that PET/CT has little value in initial imaging for early-stage NPC patients. Therefore, this expensive imaging test should be used pragmatically, and its usage should be based on well-designed prospective studies targeting specific indications. Cost-effectiveness analyses of PET/CT scans according to clinical indications are also warranted.

## Supplementary material

ESM 1 **Figure S1** ROC curves of PET/CT and head-and-neck MRI in detecting lymph node metastasis. (a) ROC curves of PET/CT and head-and-neck MRI in detecting retropharyngeal lymph node metastasis; (b) ROC curves of PET/CT and head-and-neck MRI in detecting neck lymph node metastasis. (DOCX 1133 kb)

## Abbreviations

AJCC: American Joint Committee on Cancer
CCRT: Concurrent chemoradiotherapy
CLN: Cervical lymph node
CR: Complete response
CWU: Conventional workup
DMFS: Distant metastasis-free survival
EBV: Epstein–Barr virus
ECOG: Eastern Cooperative Oncology Group
ICT: Induction chemotherapy
IQR: Interquartile range
LN: Lymph node
LRFS: Local relapse-free survival
LRRFS: Locoregional relapse-free survival
MRI: Magnetic resonance imaging
NCCN: National Comprehensive Cancer Network
NPC: Nasopharyngeal carcinoma
NPV: Negative predictive value
PD: Progressive disease
PFS: Progression-free survival
PPV: Positive predictive value
PR: Partial response
PSM: Propensity score matching
RLN: Retropharyngeal lymph node
RRFS: Regional recurrence-free survival
RT: Radiotherapy
SD: Stable disease
SOT: The standard of truth
