# Supplementary material for: Low value of whole-body dual-modality [18f]fluorodeoxyglucose positron emission tomography/computed tomography in primary staging of stage I–II nasopharyngeal carcinoma: a nest case-control study
Source: Eur Radiol. 2021 Jan 8;31(7):5222–33. doi: 10.1007/s00330-020-07478-1 (PMC8213607; doi:10.1007/s00330-020-07478-1)

**PET/CT and MRI imaging**

All patients fasted for at least 6 h before PET/CT scans. Patients’ serum glucose levels were measured and confirmed to be less than 200 mg/dL before imaging. PET/CT imaging was performed with an integrated PET/CT system (Discovery ST 16; GE Healthcare). The PET parameters were as follows: 3 min/bed position, matrix 200 × 200, field of view (FOV) 740mm, slice thickness 3mm, and full width at half maxima (FWHM) was 5 mm. The reconstruction was performed using the ordered subset expectation maximization iterative algorithm (OSEM). Helical CT was arranged from the head to the proximal thigh prior to PET acquisition, and CT parameters were set to match PET data according to a standardised protocol (120 kV; 200 mA; rotation time, 0.5 second; pitch, 1; collimation slice, 1.25mm; FOV 500mm; matrix 512*512, slice thickness 3mm). PET/CT scans from the head to the proximal thigh were started at 45 to 60 min after the administration of 5.55 MBq/kg ^18^F-FDG. The PET images were reconstructed with the use of CT data for attenuation correction and anatomical correlation.

MRI imaging was performed with a 1.5 or 3.0-T system (Signa CV/i; General Electric

Healthcare) employing the spin-echo technique. The region from the suprasellar cistern to the inferior margin at the sternal end of the clavicle was examined in each patient by using a head and neck–combined coil. T1-weighted images in the axial, coronal, and sagittal planes (repetition time: 500–600 ms, echo time: 10–20 ms, and field of view: 22 cm) and axial T2-weighted images (repetition time: 4,000–6,000 ms, echo time: 95–110 ms, and field of view: 22 cm) were obtained. After intravenous injection of the contrast material (0.1 mmol/kg gadopentetate dimeglumine; Magnevist, Schering), axial and sagittal T1-weighted images and T1-weighted fat-suppressed coronal sequences (FOV 220mm, section thickness 5 mm, matrix size 512*512) were acquired sequentially.

**Table S1** The treatment that PET/CT patients and non-PET/CT patients followed.

|  | PSM dataset | |  |
| --- | --- | --- | --- |
|  | PET/CT+CWU group  (n=218) | CWU group  (n=654) | *P* |
| Treatment |  |  | 0.334 |
| RT | 90(41.3) | 288(44.0) |  |
| CCRT | 78(35.8) | 251(38.4) |  |
| ICT+RT | 19(8.7) | 49(7.5) |  |
| ICT+CCRT | 31(14.2) | 66(10.1) |  |

Abbreviations: PSM, propensity score matching; CWU, conventional work-up; RT, Radiotherapy; CCRT, Concurrent Chemoradiotherapy; ICT, Induction Chemotherapy

P value < 0.05 indicates a statistically significant difference


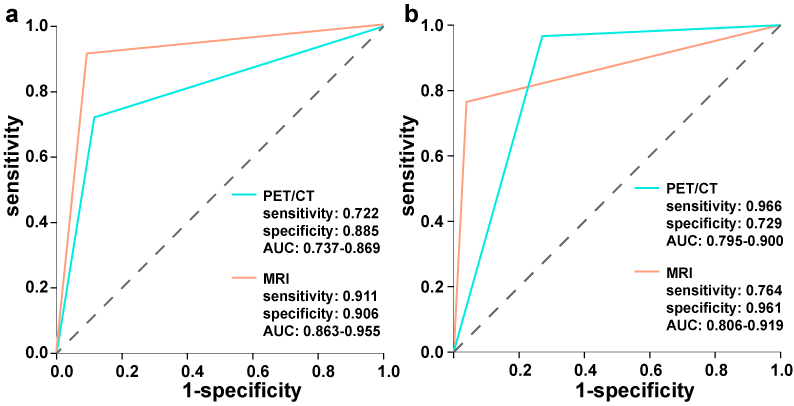

Supplement: Supplementary file 1 — Figure S1 ROC curves of PET/CT and head-and-neck MRI in detecting lymph node metastasis. (a) ROC curves of PET/CT and head-and-neck MRI in detecting retropharyngeal lymph node metastasis; (b) ROC curves of PET/CT and head-and-neck MRI in detecting neck lymph node metastasis. (DOCX 1133 kb) [file 330_2020_7478_MOESM1_ESM.docx]
